# C5a-C5aR1 Axis Activation Drives Envenomation Immunopathology by the Snake *Naja annulifera*

**DOI:** 10.3389/fimmu.2021.652242

**Published:** 2021-04-15

**Authors:** Felipe Silva de França, Isadora Maria Villas-Boas, Bruno Cogliati, Trent M. Woodruff, Edimara da Silva Reis, John D. Lambris, Denise V. Tambourgi

**Affiliations:** ^1^ Immunochemistry Laboratory, Instituto Butantan, São Paulo, Brazil; ^2^ Department of Pathology, School of Veterinary Medicine and Animal Science, University of São Paulo, São Paulo, Brazil; ^3^ Neuroinflammation Laboratory, School of Biomedical Sciences, The University of Queensland, St Lucia, QLD, Australia; ^4^ Department of Pathology and Laboratory Medicine, Perelman School of Medicine, University of Pennsylvania, Philadelphia, PA, United States

**Keywords:** *Naja* snake venom, envenomation, complement system, C5a-C5aR1, complement inhibitors

## Abstract

Systemic complement activation drives a plethora of pathological conditions, but its role in snake envenoming remains obscure. Here, we explored complement’s contribution to the physiopathogenesis of *Naja annulifera* envenomation. We found that *N. annulifera* venom promoted the generation of C3a, C4a, C5a, and the soluble Terminal Complement Complex (sTCC) mediated by the action of snake venom metalloproteinases. *N. annulifera* venom also induced the release of lipid mediators and chemokines in a human whole-blood model. This release was complement-mediated, since C3/C3b and C5a Receptor 1 (C5aR1) inhibition mitigated the effects. In an experimental BALB/c mouse model of envenomation, *N. annulifera* venom promoted lipid mediator and chemokine production, neutrophil influx, and swelling at the injection site in a C5a-C5aR1 axis-dependent manner. *N. annulifera* venom induced systemic complementopathy and increased interleukin and chemokine production, leukocytosis, and acute lung injury (ALI). Inhibition of C5aR1 with the cyclic peptide antagonist PMX205 rescued mice from these systemic reactions and abrogated ALI development. These data reveal hitherto unrecognized roles for complement in envenomation physiopathogenesis, making complement an interesting therapeutic target in envenomation by *N. annulifera* and possibly by other snake venoms.

## Introduction

Complement activation is a crucial event influencing the development of innate and adaptive immune responses (1, 2). Once microbial associated molecular patterns (MAMPs) and damage-associated molecular patterns (DAMPs) have been detected, complement can become activated through three intrinsic pathways, the alternative (AP), lectin (LP), and classical (CP) pathways, or through extrinsic pathways involving coagulation proteases, cathepsins, elastase, or snake venom metalloproteases and serine proteases (2, 3). All of these pathways converge at central events that culminate in the cleavage of C3, C4, and/or C5, leading to the generation of opsonins (C3b and C4b) and anaphylatoxins (C3a, C4a, and C5a) and assembly of the terminal complement complex (TCC; C5b-9_n_). Acting *via* various cell types and receptors, these products stimulate a number of inflammatory events, inducing mast cell degranulation (4), lipid mediator release (5–9), inflammasome assembly (10–12), chemotaxis (2), generation of ROS and NOS (13–15), and production of interleukins and chemokines (1). Although complement is essential to host defense and physiology, deficiencies or uncontrolled activation of complement components can be detrimental; complement components can promote excessive inflammation that culminates in tissue damage, organ dysfunction, permanent disabilities, and sometimes death. Such results have been observed in a myriad of inflammatory disorders (16–19), as well as in envenomation by snakes (20).

Snakebite envenoming constitutes a public health problem in tropical and subtropical countries of Africa, Asia, and Latin America. The clinical consequences of these accidents are diverse, including respiratory arrest, hemostatic disorders, bleeding, and tissue injury. Snakebites are responsible for more than 80,000 deaths per year and cause amputation or permanent disability in about 300,000 victims each year (20–23). Given the relevance of snakebite, the World Health Organization (WHO) has established a program to reduce snakebite envenoming-associated mortality and disabilities by 50% before the year 2030. This program includes incentives for studies of “next-generation” treatments (23), whose development will require optimal characterization of the molecular mechanisms involved in the physiopathogenesis of envenomation.

It is particularly notable that the snakebite envenoming process presents some of the same clinical features observed in certain complement-mediated inflammatory conditions, making this system an interesting therapeutic target. Several studies have shown that snake venom components can interact with complement proteins (3). By the last century, cobra venom factor (CVF), a C3b-like protein isolated from *Naja* venom had been fully characterized in terms of its complement-depleting activity. This venom has been shown to trigger AP activation in an exacerbated and uncontrolled manner in a number of experimental models (24–26). In addition, venoms from snakes of different genera, such as *Trimeresurus*, *Bothrops*, and *Micrurus*, have been shown to trigger complement activation in normal human serum (NHS) *in vitro*, leading to anaphylatoxin generation and soluble Terminal Complement Complex (sTCC) assembly. These events are in part associated with the action of snake venom metalloproteinases (SVMP) and snake venom serine proteinases (SVSP) on central complement components and regulators (27–33). In addition, other studies have demonstrated depletion of C3 and Factor B (34) and an increase in anaphylatoxins and sTCC plasma levels in envenomated patients, indicating complement activation (35, 36). Despite these experimental and clinical reports, the real impact of complement activation on the physiopathogenesis of envenomation by snakes remains unclear.

Recently, we have reported that *Naja annulifera* snake venom contains various potential complement activators, including CVF, SVMPs, SVSPs, and proteins containing mannose and N-acetylglucosamine residues. Furthermore, this cobra venom induces local reactions, characterized by swelling mediated by mast cell degranulation, release of lipid mediators and neutrophil infiltration, and systemic reactions characterized by an increase in plasma levels of C-C Motif Chemokine Ligand 2 (CCL2) and Interleukin 6 (IL-6), neutrophilia, monocytosis, and pulmonary damage (37). Considering these findings, we believe that *N. annulifera* venom exhibits interesting characteristics that make it useful for models to evaluate the impact of complement activation on envenomation physiopathogenesis.

Here, we have shown that *N. annulifera* venom triggers complement activation *in vitro* and *in vivo*, followed by the release of inflammatory mediators. By performing pharmacological interventions in a human whole-blood model of inflammation, we have also demonstrated that blockade of C3 or C5 cleavage and C5aR1 signaling inhibition reduce several inflammatory parameters associated with envenomation immunopathology. Furthermore, in various mouse models of envenomation, we have shown that C5a-C5aR1 axis activation is crucial for local and systemic inflammation and that changes induced by the venom, including pulmonary injury, can be abrogated by the use of PMX205, a C5aR1 antagonist. Thus, C5aR1 signaling seems to be an interesting therapeutic target in snakebite accidents involving *N. annulifera* or other snakes in which envenomation pathogenesis is driven by C5a-C5aR1 axis activation.

## Material and Methods

### *N. annulifera* Venom

*N. annulifera* snake venom (South Africa specimens) was purchased from LATOXAN Laboratory (Portes-les-Valence, France). The lyophilized venom was reconstituted in sterile saline at 5 mg/mL and stored at -80°C until use. The protein and endotoxin contents were quantified by using a BCA assay protein kit (Pierce) and PYROGENT™ Plus Gel Clot LAL Assay (Lonza, USA), respectively, according to manufacturers’ recommendations. The endotoxin was present in the venom at a level below the assay’s sensitivity (< 0.125 EU/mL).

### Complement Therapeutics

PMX205 and P32 peptides were synthesized as previously described (38, 39). Cp40 was synthesized as described by Qu et al. (40). SB290157 was obtained from Cayman Chemical (Michigan, USA).

### Ethics Statement

BALB/c male mice (18–22 g) were obtained from the Center for Animal Breeding of Butantan Institute. All procedures involving animals were carried out in accordance with the ethical principles for animal research adopted by the Brazilian Society of Animal Science and the National Brazilian Legislation n˚.11.794/08. The protocols used in the present study were approved by the Institutional Animal Care and Use Committee of the Butantan Institute (protocol approval n˚ 5323120918). Experiments conducted with human samples were approved by the Human Research Ethics Committee of the Municipal Health Secretary of São Paulo. Blood samples were obtained from healthy donors after informed consent (protocol approval n˚ 974.312 and 4.309.960).

### *In Vitro* Experiments

#### Erythrocytes and Sera

Blood from normal sheep and rabbits was collected in an equal volume of Alsever solution (citrate, 114 mM; glucose, 27 mM; NaCl, 72 mM; pH 6.1) and maintained at 4°C. Human blood samples obtained from healthy donors were collected without anticoagulant and allowed to clot at 4°C for 4 h. The blood was then centrifuged at 400 x *g*, and the normal human serum (NHS) was collected and stored at -80°C until use.

#### Complement Assays

NHS samples (200 µL) were treated with crude *N. annulifera* venom (50 µg) or sterile saline and incubated for 30 min at 37°C. The anaphylatoxins and sTCC in these samples were then quantified using BD™ CBA Human Anaphylatoxin (BD Biosciences) and MicroVue SC5b-9 Plus Enzyme Immunoassay (Quidel) Kits, respectively. AP, CP, and LP activity assays were performed as described (41, 42).

##### Direct Cleavage of Human Complement Components

Samples of purified human C3, C4, and C5 (2.5 µg each) (CompTech, Inc) were incubated with *N. annulifera* venom (2.5 µg) for 30 min at 37°C in the presence or absence of 1,10-phenanthroline (1,10 Phe) (10 mM) or phenylmethylsulfonyl fluoride (PMSF) (10 mM) SVMP and SVSP inhibitors, respectively. The reactions were stopped by adding Ethylenediamine Tetraacetic Acid (EDTA) (15 mM), and the mixtures were subjected to electrophoresis on 10% Sodium Dodecyl Sulfate Polyacrylamide Gels (SDS-PAGE) (43) under reducing conditions, and the gels were silver-stained (40). In addition, the generation of the anaphylatoxins was quantified using the BD™ Cytometric Bead Array (CBA) Human Anaphylatoxin kit (BD Biosciences).

#### *Ex-Vivo* Human Whole-Blood Model

The ex-vivo human whole-blood model described by Mollness and colleagues (44) was used, with some modifications. Blood samples were collected by venipuncture into tubes containing 50 µg/mL of lepirudin (Refludan^®^, Colgene, USA), a thrombin-inhibitor anticoagulant, which does not interfere with complement activity. The whole-blood samples were treated with increasing concentrations of *N. annulifera* venom, ranging from 3.125 to 100 µg/mL, or with sterile saline and then incubated for 30 or 60 min at 37°C. Under these conditions, *N. annulifera* venom induces the production of inflammatory mediators but does not promote coagulation; however, at higher concentrations (50 and 100 µg/mL), *N. annulifera* venom is highly hemolytic. Considering that free hemoglobin may be highly inflammatory (45) and that there is no information about hemolysis on envenomation by *N. annulifera*, we chose 25 µg/mL for subsequent experiments, since this dose did not induce significant hemolysis (data not shown).

To assess complement’s role in the inflammatory events promoted by *N. annulifera* venom, human whole blood was pretreated with either the compstatin analog Cp40 (C3/C3b inhibitor, 20 µM) (46), SB290157 (C3aR antagonist, 20 µM) (47), PMX205 (C5aR1 antagonist, 20 µM) (48) or P32 (C5aR2 agonist, 100 µM) (39) inhibitors/activator or with appropriate vehicle as control, *i.e*., saline (Cp40 and P32), DMSO (SB290157), or 5% glucose (PMX205), for 5 min at room temperature. In addition, *N. annulifera* venom samples were incubated with 1,10 Phe (SVMP inhibitor, 15 mM) or vehicle (ethanol) for 15 min at room temperature. *N. annulifera* venom was then added to the blood samples, and the mixtures were incubated at 37°C for 30, 60, or 120 min under continuous agitation. Finally, the tubes were centrifuged at 405 × *g*, for 10 min at 4°C, the plasma was collected, and the samples were stored at -80°C for further quantification of inflammatory mediators.

### *In Vivo* Experiments

#### Analysis of Systemic Complement Activity

BALB/c mice (n=6/group) were injected by the intraperitoneal (i.p.) route with a sublethal (56.5 µg) or lethal (94.2 µg) dose of *N. annulifera* venom to induce moderate or severe envenomation illness, respectively (37). As controls, mice were inoculated with sterile saline. After 30 min, all mice were euthanized with an overdose of two anesthetics (200 mg/kg ketamine, 20 mg/kg xylazine). The whole blood was collected by cardiac puncture and allow to clot at room temperature for 1 hr. The blood samples were then centrifuged at 1500 × *g* for 15 min at 4°C, and the sera obtained were stored at -80°C until analysis. Measurements of AP, CP, and LP serum activity were evaluated at level of C9 by using the Complement Pathway Mouse Assay ELISA kit (HycultBiotech) according to the manufacturer’s instructions. The results were expressed as AP, CP, or LP activity [%].

#### Assessment of the C5a-C5aR1 Axis Contribution to *In Vivo* Reactions

Mice were treated by the subcutaneous (s.c.) route with PMX205 (C5aR1 antagonist) or vehicle (5% glucose solution) at 1 or 2 mg/kg b.wt. at 24 h or 1 h before the induction of local and systemic reactions.

#### Local Reactions

BALB/c mice (n=6/group) were each injected with 10 μg of *N. annulifera* venom (37), dissolved in a volume of 50 μL sterile saline, into the s.c. tissue of the plantar region of the left hind paw. The contralateral hind paw (control) was inoculated with 50 μL of sterile saline. The thickness of the hind paws was assessed with a caliper rule (Mitutoyo, Suzano-SP, Brazil; in increments of 0.01 mm) at various time points before injection (T0) and after 24 h, following inoculation with either venom or saline (Te). Increases in paw volume were expressed as a percentage (%), calculated according to the following formula: (Te-T0)/T0*100 (37). Mice were euthanized by anesthetic overdose (200 mg/kg ketamine, 20 mg/kg xylazine) at 20 or 60 min after venom inoculation, before collection of the s.c. tissue from hind paws for inflammatory mediator quantification. Tissue samples were homogenized using a PT-10 Polytron homogenizer (Kinematica, Luzern, SWZ) in lysis buffer (NaCl, 200 mM; EDTA, 5 mM; Tris, 10 mM; glycerol, 10%; leupeptin, 1 µg/mL; aprotinin, 28 µg/mL; PMSF, 1 mM). The homogenized tissues were then centrifuged at 1500 × g for 15 min at 4°C. The supernatants were obtained, centrifuged again, filtered, and stored at -80°C until analyzed.

#### Systemic Reactions

The C5a-C5aR1 contribution to envenomation systemic reactions were scrutinized by sublethal and lethal experimental sets which represents moderate and severe envenomation conditions, respectively (37). In the moderate set mice were inoculated by the i.p. route with a sublethal *N. annulifera* venom dose (37) or with sterile saline and euthanized 1 h after venom injection with anesthetics overdose (200 mg/kg ketamine, 20 mg/kg xylazine) to blood samples obtention. Blood and lung samples from mice submitted injected with the venom lethal dose (37) were obtained 5 hours after venom inoculation which represents death moment or intense impairment of the animals this group. The animals that not died at 5 hours after venom injection were euthanized with anesthetics overdose (200 mg/kg ketamine, 20 mg/kg xylazine), since this period was determined as endpoint to this group. Blood samples were then obtained by cardiac puncture using EDTA (2.5 mg/mL) as an anticoagulant. Aliquots of these samples were used for systemic total and differential leukocyte counts, and other blood samples were centrifuged at 2800 × g at 4°C for 10 min. Plasma samples were stored at -80°C and used to measure inflammatory mediators.

After euthanasia, the lungs were extracted from the mice injected with the lethal *N. annulifera* venom dose, then fixed in 10% formaldehyde for 24 h. The pulmonary samples were then subjected to routine histologic fixation and stained with hematoxylin and eosin (HE). The tissue samples were examined under a light microscope for the presence of cellular/tissue changes, and a histopathological score was determined.

#### Quantification of Inflammatory Mediators

Human chemokines were detected by using a BD™ CBA Human Chemokine kit (BD Biosciences). The BD™ CBA Mouse Inflammation kit (BD Biosciences) was used to detect systemic chemokines and interleukins in the mouse plasma. Keratinocyte Chemokine (KC)/C-X-C motif chemokine ligand 1(CXCL1) was detected by using the LEGENDplex™ Mouse Anti-Virus response panel (Biolegend). Mouse myeloperoxidase (MPO) was quantified by using a MPO Mouse ELISA kit (HycultBiotech). leukotriene B4 (LTB_4_), prostaglandin E2 (PGE_2_), and thromboxane B2 (TXB_2_) were quantified by the LTB4 Enzyme-Linked Immunosorbent Assay (ELISA), PGE2 Monoclonal, and TXB2 ELISA kits, all from Cayman Chemical. All assays were performed according to the manufacturer’s recommendations.

### Statistical Analysis

Statistical analysis was performed using Student’s *t-*test for comparisons of the mean of two groups. One-way and two-way ANOVA, followed by Bonferroni’s multiple comparison test, were applied to the results of the time and dose-response experiments. The statistical analyses were conducted using Graphpad Prism 5 software (La Jolla, California, USA). Differences were considered significant when p ≤ 0.05.

## Results

### *N. annulifera* venom acts on the human complement

**Figure 1:**
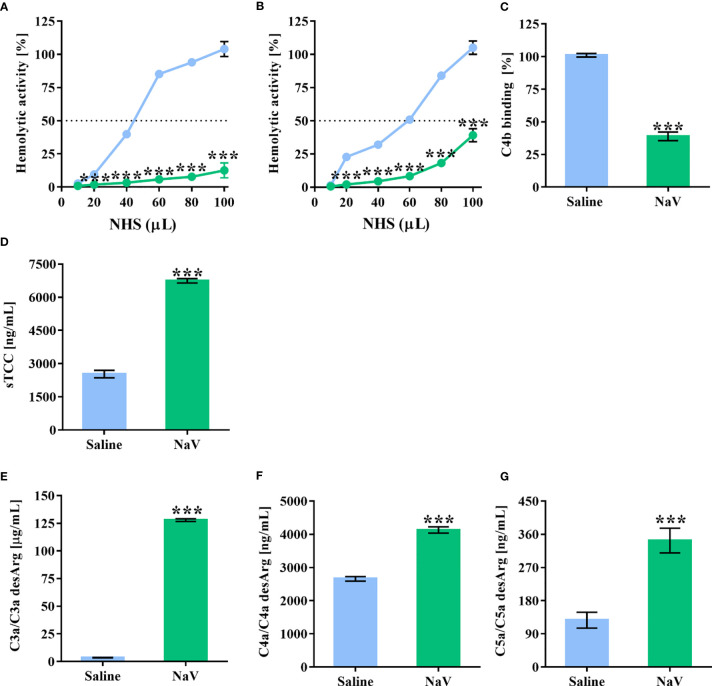
*N. annulifera* venom (NaV) acts on the human complement system. NHS samples were incubated with NaV or sterile saline for 30 minutes at 37°C. Then, these samples were submitted to functional assays to determine NaV effects on AP **(A)**, LP **(B)** and CP **(C)** activity. In addition, C3a **(D)**, C4a **(E)** and C5a **(F)** anaphylatoxins generation, and sTCC **(G)** assembly were measured by ELISA. Data represent means ± SEM of five independent experiments from different NHS donors. ***p ≤ 0.001 (two-tailed *t*-test or two-way ANOVA, followed by Bonferroni post-test).

In a previous study (37), we demonstrated the presence of several potential components in *N. annulifera* venom able to interact with the human complement system. To determine whether *N. annulifera* venom could interfere with the complement activity, NHS samples were incubated with the venom and then submitted to hemolytic (AP and CP) and C4b (LP) ELISA assays (41, 42). We found that cobra venom significantly reduced the activity of the three complement pathways (**Figures 1A–C**). In order to evaluate whether the reduction in complement activity was a result of activation or inhibition, since some animal venoms and secretions can contain complement inhibitors (3), we assessed anaphylatoxin generation and sTCC assembly. The results (**Figures 1D–G**) confirmed that venom promoted complement activation in NHS, as determine by the generation of C3a, C5a, and sTCC, and to a lesser extent C4a.

### SVMP and SVSP Hydrolyze Human Complement Proteins

**Figure 2:**
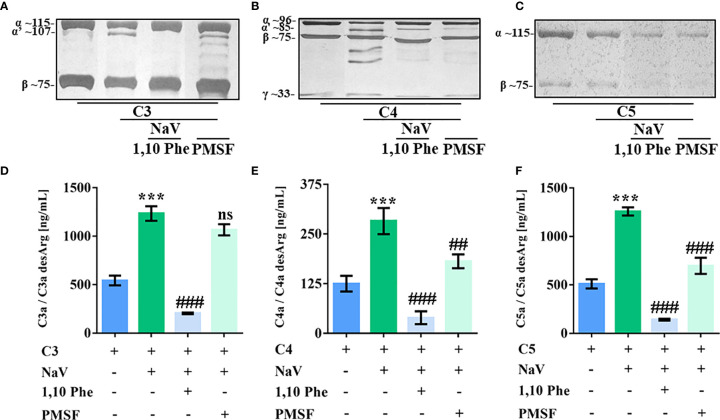
SVMP and SVSP cleave human complement proteins. Human C3, C4 and C5 purified proteins (2.5 µg) were incubated with sterile saline or *N. annulifera* venom (NaV - 2.5 µg) with or without metallo- (10 mM) and serine-proteinases (10 mM) inhibitors for 30 minutes at 37°C. After this period, reactions were stopped and C3 **(A)**, C4 **(B)** and C5 **(C)** cleavage evaluated by SDS-PAGE and silver stanning. Images presenting in panels a-c were grouped and/or spliced (the original gels are presented as supplementary material – **Figure S5**). C3a **(D)**, C4a **(E)** and C5a **(F)** anaphylatoxins generation in these reactions were determined by CBA. Panels **(A–F)** represents five different experiments. Data are means ± SEM of three independent experiments ***p ≤ 0.001 (two-tailed *t*-test). ^###^ indicates a significant difference between complement purified proteins treated with *N. annulifera* venom + Vehicle and *N. annulifera* venom + Protease inhibitors. ^##^p ≤ 0.01, ^###^p ≤ 0.001. ns = non-significant.

SVMP and SVSP present many substrates and actions on prey and human victims (49), making them potential complement activators. By incubating purified human complement proteins with *N. annulifera* venom and using specific inhibitors of metalloproteases and serine proteases, we observed that both classes of enzymes present in the venom were able to cleave C4 and C5, while C3 is cleaved only by SVMP (**Figures 2A–C**). In addition, C3, C4 and C5 cleavage by venom proteases was functional, since culminated in the C3a, C4a and C5a anaphylatoxins generation (**Figures 2D–F**).

### *N. annulifera* Venom Induces Inflammation *In Vitro*

The *N. annulifera* venom inflammatory potential was tested in a human whole-blood *ex vivo* model in the presence of lepirudin, a thrombin inhibitor anticoagulant that does not interfere with complement activity (44, 45). Human blood samples were incubated with increasing *N. annulifera* venom concentrations (3.125 - 100 µg/mL) for 30 or 60 min at 37°C, and the production of inflammatory mediators was quantified. We found that *N. annulifera* venom triggered anaphylatoxin generation and sTCC assembly (**Supplementary Figure 1**); these processes were accompanied by the release of lipid mediators, including leukotriene B_4_ (LTB_4_), prostaglandin E_2_ (PGE_2_), and thromboxane A_2_ (TXA_2_) (**Supplementary Figure 2**). Furthermore, *N. annulifera* venom induced the production of the chemokines C-C motif chemokine ligand 2 (CCL2), CCL5 and C-X-C motif chemokine ligand 8 (CXCL8) (**Supplementary Figure 3**).

### Inflammatory Events Induced by *N. annulifera* Venom *In Vitro* Are Complement-Mediated

**Figure 3:**
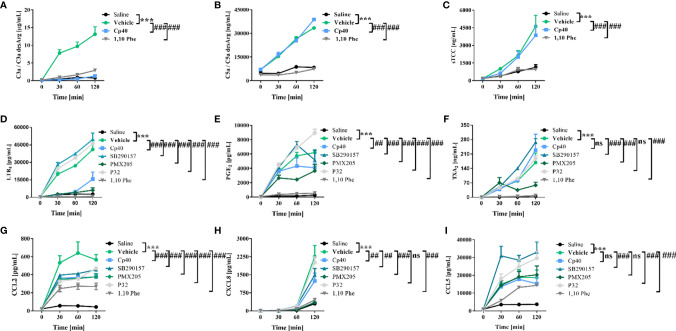
Human complement activation by *N. annulifera* venom (NaV) results in the generation of chemokines and lipid mediators. Human whole-blood samples were incubated for 5 minutes, at room temperature, with Cp40 (C3 cleavage inhibitor), SB290157 (C3aR antagonist), PMX205 (C5aR1 antagonist), P32 (C5aR2 agonist) or 1,10 Phenanthroline (metalloproteinase inhibitor) inhibitors, or their respective vehicles. Then, all these samples were exposed to NaV or sterile saline during 30, 60 and 120 minutes, and complement activation products generation **(A–C)**, lipid mediators release **(D–F)** and chemokines upregulation **(G–I)** scrutinized by CBA or ELISA. Data are means ± SEM of six independent experiments with different whole-blood donors. ***p ≤ 0.001 (two-tailed two-way ANOVA, followed by Bonferroni post-test). ^###^ indicates a significant difference between *N. annulifera* venom + Vehicle and *N. annulifera* venom + Inhibitors. **(E, H)** ^##^ means the comparison between samples exposed to the NaV + vehicle and NaV+ complement inhibitors in which statistical differences are p ≤ 0.01. ns = non-significant.

To identify the contribution of complement to the inflammatory reactions promoted by *N. annulifera* venom, we characterized the effects of pharmacologic interventions on a human *ex vivo* whole-blood model. The compstatin analog peptide Cp40, a C3/C3b inhibitor (46), strongly reduced C3a (**Figure 3A**) and LTB_4_ (**Figure 3D**) production and to a lesser extent that of PGE_2_ (**Figure 3E**), CCL2 (**Figure 3G**), and CXCL8 (**Figure 3I**). In contrast, Cp40 failed to interfere with the generation of C5a or sTCC assembly (**Figures 3B, C**), suggesting that *N. annulifera* venom proteases actions upon C5 is responsible for it (**Figures 2C, F**). The incubation of *N. annulifera* venom with 1,10 Phe, a SVMP inhibitor, and further incubation with human whole blood resulted in the abrogation of C3a/C5a generation and sTCC formation (**Figures 3A–C**), suggesting an important role for SVMP in complement activation induced by *N. annulifera* venom in this *ex vivo* model. Since *N. annulifera* venom proteases can lead to complement activation, and Cp40 failed to control the generation of C5a or sTCC, we decided to evaluate the role of anaphylatoxin receptors in the inflammatory process induced by cobra venom.

Inhibition of C3a receptor (C3aR) by the antagonist SB290157 (47) resulted in a reduction in CXCL8 and CCL2 production (**Figures 3G, H**) and an increased release of lipid mediators (**Figures 3D–F**) and CCL5 (**Figures 3I**). In contrast, C5a receptor 1 (C5aR1) inhibition caused by the antagonist PMX205 (48) resulted in decreased levels of LTB_4_, PGE_2_, TXA_2_, CXCL8, and CCL2 (**Figures 3D–H**).

C5a receptor 2 (C5aR2), a second receptor that binds C5a, has been described to act as an immune dampener to C5aR1 and TLR-4, regulating the production of inflammatory mediators (50). To test if C5aR2-mediated signaling could modulate the release of inflammatory markers in our model, a functionally selective agonist peptide (P32) (39) was used. P32 reduced CCL2 production to the same extent as did the other inhibitors (**Figure 3G**). On the other hand, C5aR2 stimulation by P32 potentiated LTB_4_, PGE_2_ (**Figures 3D, E**) and CCL5 release (**Figure 3I**).

Given that *N. annulifera* venom triggers complement activation by the extrinsic route and that the modulation of the C5a-C5aR1 axis interferes with the immunopathology associated with envenomation *in vitro*, we chose PMX205 for evaluating complement’s contribution to local and systemic reactions in murine models of envenomation.

### *N. annulifera* Venom Promotes Systemic Complementopathy

**Figure 4:**
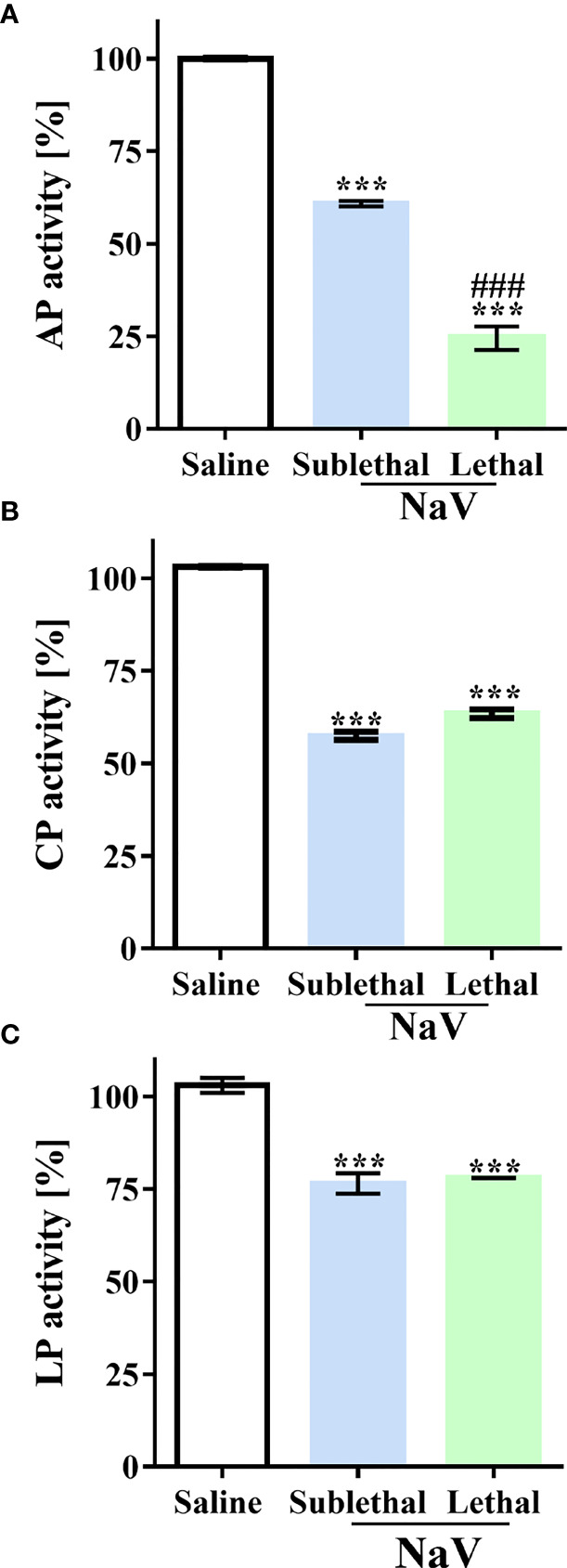
*N. annulifera* venom (NaV) induces systemic complementopathy in the murine model. Serum samples were obtained from mice envenomated, i.p., with a sublethal or lethal NaV dose (n=6/venom dose) and AP **(A)**, LP **(B)** and CP **(C)** activity evaluated by functional assays. Data are means ± SEM of six independent experiments. ***p ≤ 0.001 (two-tailed one-way ANOVA, followed by Bonferroni post-test). ^###^ indicates significant difference between the sublethal and lethal venom doses.

Previously, we showed that *N. annulifera* venom can induce systemic inflammatory reactions in mice (37). Nevertheless, action on the complement system in this scenario had not been evaluated. To examine this parameter, we injected groups of mice with sublethal or lethal (37) doses of venom by the intraperitoneal (i.p.) route; control animals were injected with sterile saline. Blood samples were obtained after 30 min, and complement activity was determined by functional assays at the level of C9 activation using mouse serum samples. Moderated and severe experimental envenoming induced CP and LP complementopathy at the same extent (**Figures 4B, C**). In both experimental groups, AP activity reduction was detected, however, in mice submitted to the lethal envenomation protocol the AP consumption was higher (**Figure 4A**).

### C5a-C5aR1 Axis Modulation Reduces Local Reactions Induced by *N. annulifera* Venom

**Figure 5:**
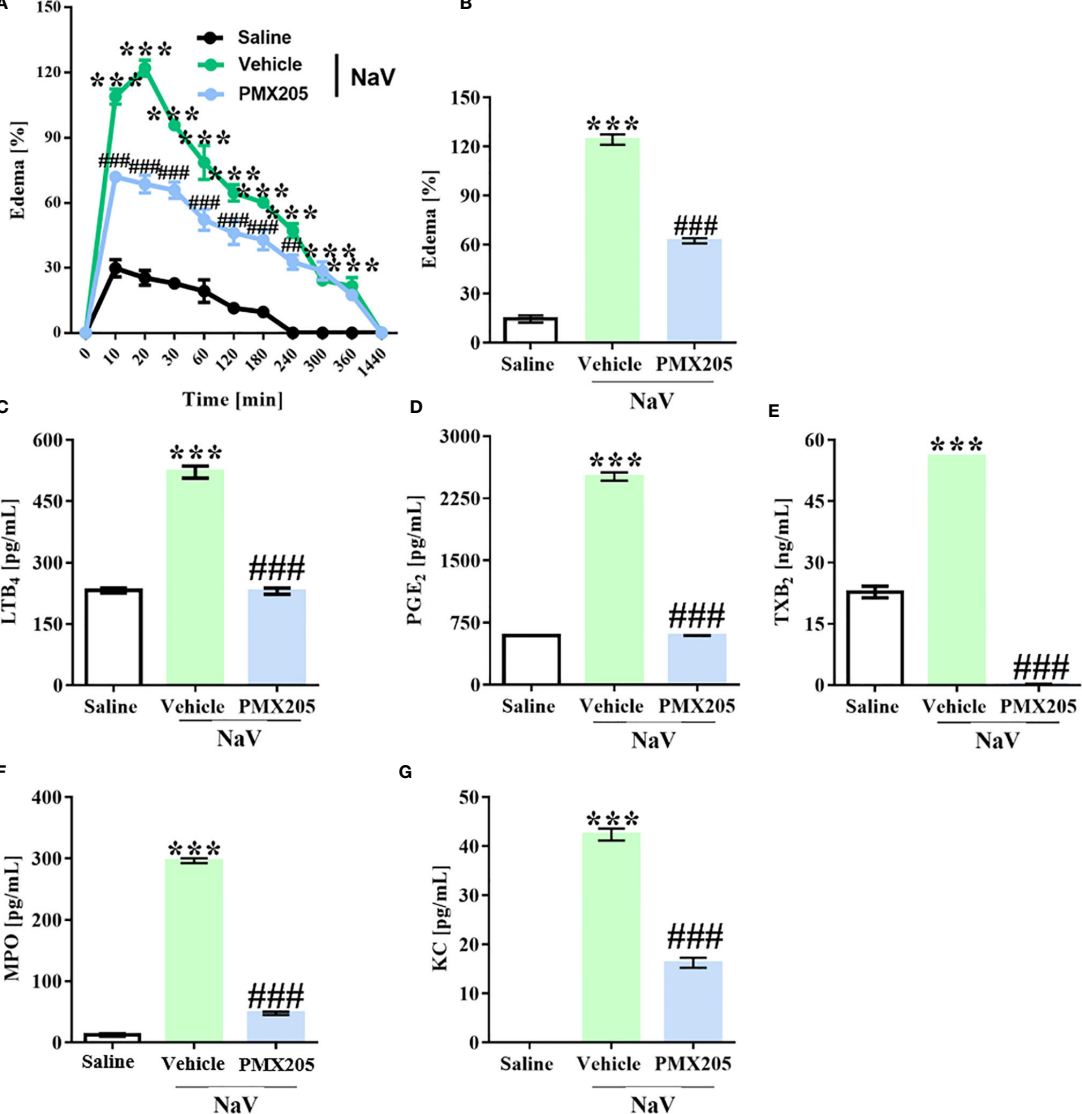
*N. annulifera* venom (NaV) promotes C5a-C5aR1 axis-dependent edema. Mice (n=6/group) were pretreated with PMX205 (1 mg/kg), a C5aR1 inhibitor, or vehicle 24 and 1 hour before NaV injection. Following inhibitor administration, local reactions were induced by NaV inoculation into subcutaneous tissue from mice left hind paws and the swelling evaluated along the time with a caliper rule **(A, B)**. In addition, tissue samples were obtained along the time, homogenized, and then submitted to LTB_4_ **(C)**, PGE_2_ **(D)**, TXB_2_ **(E)**, MPO **(F)** and KC **(G)** levels assessment by ELISA and CBA. Data are means ± SEM of six independent experiments. ***p ≤ 0.001 (two-tailed one-way ANOVA or two-way ANOVA, followed by Bonferroni post-test). ^###^ indicates a significant difference between animals treated with *N. annulifera* venom + Vehicle and *N. annulifera* venom + PMX205. The ^##^ symbol (240 minutes period) means the comparison between animals treated with the vehicle + *N. annulifera* venom and PMX205 + *N. annulifera* in which statistical differences are p ≤ 0.01.

Groups of mice were treated with vehicle or PMX205 (1 mg/kg body weight [b.wt.] in 5% glucose solution) at 24 h and 1 h before *N. annulifera* venom injection. The subcutaneous venom inoculation (10 µg) induced a rapid-onset edema in the hind paws of the mice, reaching a maximum peak at 20 min after injection (125% increase in paw volume, **Figure 5A**). The swelling persisted for several hours and disappeared within 24 h. Inhibition of C5aR1 signaling produced a significant decrease in the hind paw volume with time, mainly at edema peak (54% inhibition) (**Figure 5B**). Mechanistically, the edema reduction was characterized by a strong inhibition of LTB_4_, PGE_2_, and TXA_2_ release (**Figures 5C–E**) at the edema peak.

*N. annulifera* venom injection also promoted KC (murine CXCL8 homolog) chemokine release, which was accompanied by a large neutrophil infiltration, as demonstrated by MPO quantification, that occurred in a C5a-C5aR1 axis-dependent manner, since receptor blockage reduced these inflammatory events (**Figures 5F, G**).

### C5aR1 Blockage Abrogates Systemic Reactions Evoked by *N. annulifera* Venom

**Figure 6:**
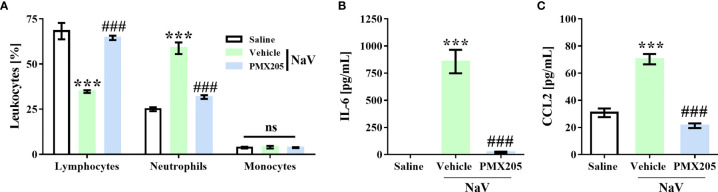
C5a-C5aR1 signaling drives moderate systemic reactions induced by *N. annulifera* venom (NaV). Mice (n=6/group) were pretreated with PMX205 (1 mg/kg), a C5aR1 inhibitor, or vehicle 24 and 1 hour before NaV injection. Following inhibitor or vehicle administration, moderate systemic reactions were induced by the injection of NaV sublethal dose, *via* intraperitoneal route. One hour after envenomation, blood samples were obtained by cardiac puncture to determine hematological changes by blood smear **(A)**, and CCL2 **(B)** and IL-6 **(C)** systemic generation by ELISA and CBA. Data are means ± SEM of six independent experiments. ***p ≤ 0.001 (two-tailed t-test or two-way ANOVA, followed by Bonferroni post-test). ^###^ indicates a significant difference between animals treated with NaV + Vehicle and NaV + PMX205. ns = non-significant.

To demonstrate the contribution of complement to the systemic reactions induced by *N. annulifera* venom, we employed two sets of experiments. The first set was performed by injecting a sublethal dose of venom (56.5 µg; i.p.), which induces a clinical condition in mice characterized by apathy, bending of the column, a rough hair coat, dyspnea, and difficulty ambulating. This dose also promotes changes in some systemic parameters, including a decrease in circulating lymphocytes and an increase in neutrophils. It also promotes increases in IL-6 and CCL2 plasma levels. All the reactions induced by this dose reached a peak 1 h after venom inoculation, and the values returned to normal by 24 h (37). This dose was not able to induce death or organ damage during any evaluation period (37). Treatment with PMX205 (1 mg/kg b.wt.) was able to restore the normal percentage of circulating lymphocytes and decrease the number of neutrophils (**Figure 6A**). Furthermore, C5aR1 inhibition fully reduced IL-6 production and restored CCL2 to physiological levels (**Figures 6B, C**).

**Figure 7:**
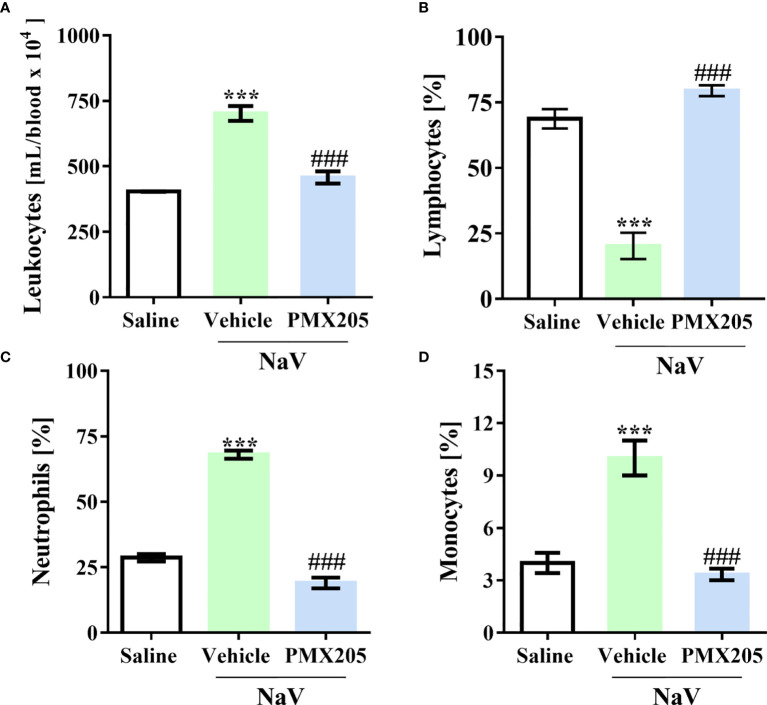
C5a-C5aR1 activation cause hematological changes in severe experimental envenomation. Mice (n=6/group) were pretreated with PMX205 (2 mg/kg), a C5aR1 inhibitor, or vehicle 24 and 1 hour before *N. annulifera* venom (NaV) injection. Following inhibitor administration, severe systemic reactions were induced by the injection of NaV lethal dose, *via* intraperitoneal route. Five hours after envenomation, blood samples were obtained by cardiac puncture to determine leukocytosis **(A)**, by cell counting in a hemocytometer, and lymphopenia **(B)**, neutrophilia **(C)**, and monocytosis **(D)**, *via* blood smear analysis. Data are means ± SEM of six independent experiments. ***p ≤ 0.001 (two-tailed t-test or two-way ANOVA, followed by Bonferroni post-test). ^###^ indicates a significant difference between animals treated with NaV + Vehicle and NaV + PMX205.

**Figure 8:**
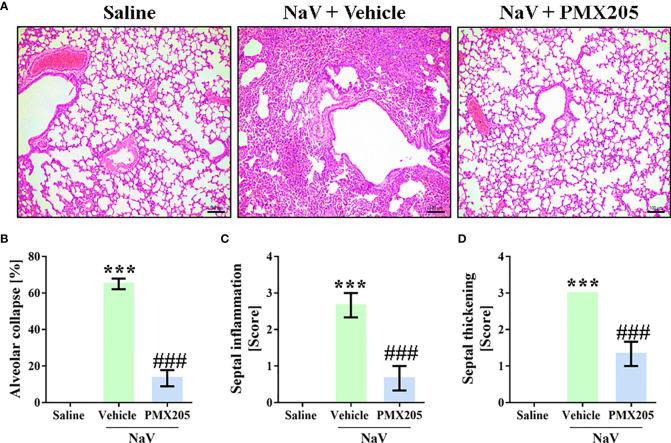
C5aR1 signaling cause ALI development in severe experimental envenomation by *N. annulifera*. Mice (n=6/group) were pretreated with PMX205 (2 mg/ kg), a C5aR1 inhibitor, or vehicle 24 and 1 hour before of *N. annulifera* venom (NaV) injection. Following inhibitor or vehicle administration, severe systemic reactions were induced by the injection of NaV lethal dose, via intraperitoneal route. Five hours after envenomation, the lungs were obtained, fixed, and submitted to histologic procedures **(A)** Results were expressed as area of alveolar collapse (%) **(B)** and histopathological scores **(C, D)**, i.e., 1- mild changes; 2- moderate changes; 3- severe/intense changes. Data are means ± SEM of six independent experiments. ***p ≤ 0.001 (two-tailed t-test or two-way ANOVA, followed by Bonferroni posttest). ^###^ indicates a significant difference between animals treated with NaV + Vehicle and NaV + PMX205.

The second experimental set was performed by injecting mice with a dose of venom equivalent to the 1LD_50_ (94.2 µg, i.p.) (37). This treatment produced a more severe clinical condition than did the sublethal dose. Mice given the higher dose showed apathy, bending of the spinal column, a rough hair coat, dyspnea, and difficulty ambulating, and they died approximately 5 h after venom inoculation. Death was preceded by systemic inflammation, characterized by leukocytosis with lymphopenia, neutrophilia, and monocytosis (**Figures 7A–D**). Moreover, the LD_50_ venom dose induced higher levels of IL-6, CCL2, and TNF-α production than did the sublethal venom dose (**Supplementary Figures 4A–C**). In the lethal context, *N. annulifera* venom induced acute lung injury (ALI), with diffuse alveolar damage (DAD) featured by alveolar collapse, septal inflammation, and a thickening of the alveolar septum (**Figures 8A–D**). Use of PMX205 (2 mg/kg b.wt.) in these mice restored the blood cell parameters to physiological levels (**Figures 7A–D**). Strikingly, ALI was completely abrogated by PMX205 treatment, along with an increase in systemic levels of an anti-inflammatory cytokine, IL-10 (**Supplementary Figure 4D**). Nevertheless, in these lethal conditions, IL-6, Tumor Necrosis Factor α (TNF-α), and CCL2 production were still aggravated (**Supplementary Figures 4A–C**).

## Discussion

Current understanding indicates that snake venoms trigger complement activation in humans (27–36), but the overall molecular mechanisms induced by the envenomation have remained underexplored. Here, by coupling *in vitro* and *in vivo* approaches, we have determined that *N. annulifera* venom induces reduction in complement pathways activity, anaphylatoxins generation and sTCC assembly. These reactions were accompanied by lipid mediators release and production of chemokines and interleukins. C5a-C5aR1 axis signaling is the driver of these effects, since its modulation prevents local and systemic reactions induced by the venom and protects mice against an extensive ALI.

Complementopathy is a hallmark of a plethora of inflammatory, autoimmune, and degenerative conditions and is generally accompanied by an increase in the systemic levels of inflammatory mediators (51–56). A human whole-blood model of inflammation has been developed to study complement’s role in various inflammatory events and diseases, including sepsis (44) and hemolytic diseases (45). This experimental model permits the investigation of the role of complement in the complex inflammatory network, including all potential cellular and fluid-phase mediators present and able to interact simultaneously. We found that *N. annulifera* venom induced complement activation in the whole-blood model, with consequent release of pro-inflammatory mediators that included LTB_4_, PGE_2_, TXA_2_, CCL2, and CXCL8. These findings are in line with the increased plasma levels of inflammatory markers observed in an *in vivo* model of envenomation, indicating that human envenomation by *N. annulifera* leads to uncontrolled inflammatory reactions and consequent development of ALI/ARDS (acute respiratory distress syndrome), a known cause of respiratory arrest and death, since patients with this inflammatory condition present augmented plasma and pulmonary levels of these pro-inflammatory mediators (57–60).

The imbalanced release of inflammatory mediators contributes significantly to envenomation pathology, since it promotes endothelial dysfunction, edema formation (61–65) pain, and tissue hypoxia, which can culminate in compartment syndrome (66–69). We have previously demonstrated that the use of eicosanoid inhibitors (37) reduces the edema induced by *N. annulifera* venom, but the underlying mechanisms have not been fully elucidated. Similar to observations in the *ex-vivo* human whole-blood model, injecting *N. annulifera* venom in the mouse subcutaneous tissue, we have demonstrated that C5a-C5aR1 axis activation was involved in the release of LTB_4_, PGE_2_, and TXA_2_ into the hind paws; these mediators are likely to be responsible for the development of the extensive swelling promoted by the venom. LTB_4_, PGE_2_, and TXA_2_ are lipid mediators that are mainly involved in vascular changes during the early stages of the inflammatory reaction (70). Nonetheless, if not controlled, the action of these mediators can be injurious, making them important players in various pathologies (70) such as in pulmonary edema and death promoted by *Tityus serrulatus* scorpion envenomation (71, 72).

Apart from swelling, *N. annulifera* venom promotes the infiltration of neutrophils into the venom inoculation site (37). C5a, LTB_4_, and CXCL-1/IL-8 are powerful chemoattractants for neutrophils (73, 74) and they have been pointed to as orchestrators in various pathological conditions. In our experiments, pharmacological inhibition of C5aR1 resulted in the abrogation of neutrophil infiltration and a decreased production of LTB_4_ and KC. Previously, we have shown that C5aR1 targeting by antibodies prevents the infiltration of neutrophils into the peritoneal cavity of mice injected with *Bothrops asper* snake venom or its purified metalloprotease (75). However, whether the C5a-C5aR1-neutrophil triad in the snake envenomation context is destructive or protective remained to be explored.

An imbalance in the systemic levels of inflammatory mediators, including C3a, C5a, and sTCC, can potentially evolve to cause multiple organ dysfunction and death (17). Injection of BALB/c mice with a sublethal venom dose, which promotes a moderate envenomation state (37) has demonstrated that *N. annulifera* venom interfere in the complement activity, culminating in the generation of C5a, which binds to C5aR1 and promotes an increase in the number of circulating neutrophils as well as IL-6 and CCL2 systemic levels. Although systemic inflammation was induced by this dose, no tissue damage was detected in the evaluated organs (brain, lungs, heart, kidneys, liver, and spleen) (37); however, this assessment does not exclude changes at the physiological level, since C5a binding to C5aR1 leads to blood pressure alteration (76), electrophysiological changes (77), pain (78), and hemostatic disorders (17, 79, 80). Thus, studies targeting these physiological parameters need to be performed.

Among the consequences of the systemic and intrapulmonary complement activation is the development of ALI/ARDS (76, 81–85), a severe form of hypoxemic respiratory failure resulting from inflammatory insult to the lungs (53–55, 86, 87). One LD_50_ injection in mice produced a severe envenomation state, with a significant reduction of the complement activity, leukocytosis, neutrophilia, monocytosis, and strong systemic production of IL-6, CCL2, and TNF-α. Furthermore, this venom dose promoted extensive ALI and death. These changes induced in mice by injection of a lethal *N. annulifera* venom dose, coupled with the *in vitro* results we obtained from the human whole-blood model, suggest that the physiopathogenesis of envenomation by *N. annulifera* is similar to that occurring in sepsis and ALI/ARDS (17, 53–55, 86, 87). In addition, strong complementopathy and increased production of interleukins and chemokines induced by the *N. annulifera*’s venom lethal dose may be related with patients’ poor prognosis, since these inflammatory events are risk factors to ALI/ARDS severity, multiorgan failure and death in sepsis and polytrauma (53, 88, 89).

The disruption of the C5aR1 signaling by the action of the PMX205 led to an increase of the anti-inflammatory IL-10 cytokine systemic levels, rescued mice from the increase on circulating leukocytes and abrogated ALI development. Unfortunately, the blockage of C5aR1 activation and the increase in IL-10 levels were not followed by a decrease of IL-6, CCL2, or TNF-α in the plasma levels, and they could not control animals’ death, induced by *N. annulifera* venom lethal dose, suggesting that perhaps other complement/inflammatory-mediated signaling pathways are acting in this context. By injecting a lethal venom dose, the physiological imbalance and tissue damage were stronger promoting high DAMPs release and amplifying complement activation. C3 is the most abundant circulating complement protein (90), and we must consider its involvement on cytokines release potentiation in lethal context, as well in the death, since *N. annulifera* venom presents on its composition various C3 activators, including CVF (37). In addition, through *in vivo* C5aR1 antagonism, C3aR activation can potentially stimulate the increase on systemic levels of inflammatory mediators (4, 11, 91–93) and promote pathological events, which can evolve to cause multiple organs dysfunction and death (53, 93–95). In addition, cobra venom also induces the formation of high amounts of sTCC, a complex with inflammatory and deleterious properties, which is a risk factor to multiple pathologies (17, 53, 96–100). Thus, by using other pharmacological tools to inhibit additional steps of the complement cascade, *in vivo*, our results may be expanded.

In recent decades, the contribution of complement to a plethora of inflammatory and degenerative diseases has been demonstrated and, in this context, various strategies to control complement activation have emerged. Complement inhibition, achieved by using eculizumab, a humanized monoclonal antibody that prevents C5 activation and improve diseases outcomes (19), has been used to treat autoinflammatory (*e.g*., atypical hemolytic uremic syndrome and paroxysmal nocturnal hemoglobinuria) and autoimmune conditions (e.g., myasthenia gravis). Thus, considering that eculizumab is an FDA-approved medicine that is already used in the clinic and that *N. annulifera* venom induces a pathology mainly mediated by C5a, eculizumab may prove useful as a complementary treatment for this envenomation. In addition, PMX205 and Avacopan (CCX168), which are C5aR1 antagonists are also under clinical development for amyotrophic lateral sclerosis (38) and atypical uremic hemolytic syndrome (aUHS), anti-neutrophil cytoplasmic antibody associated (ANCA) vasculitis, and immunoglobulin A (IgA) nephropathy (19), respectively, thus also representing other potential therapeutic medicines for envenomation treatment.

**Figure 9:**
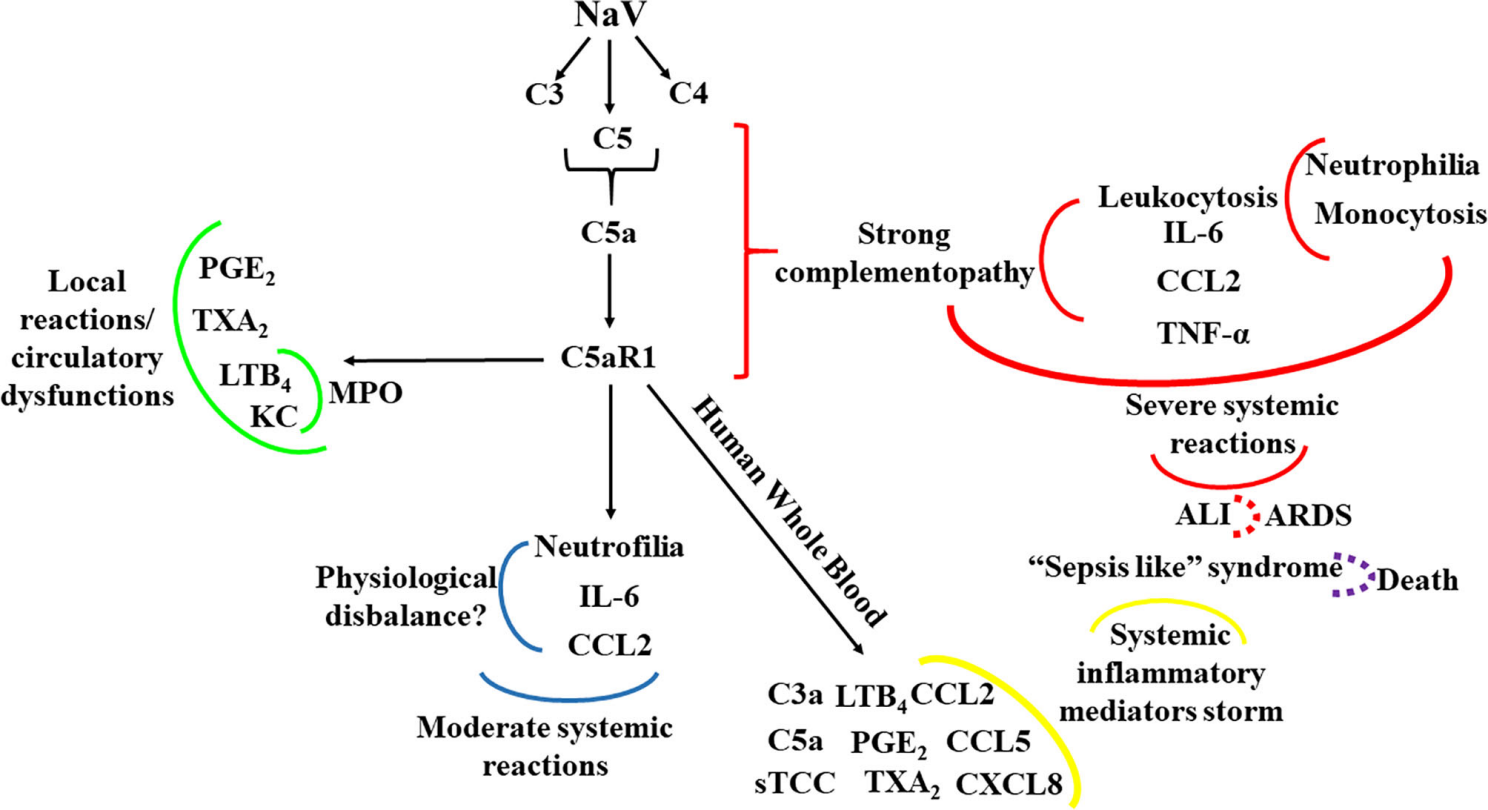
Schematic representation of the contribution of the C5a-C5aR1 axis to local and systemic reactions induced by *N. annulifera* venom (NaV). NaV acts on complement proteins culminating in anaphylatoxins generation and sTCC assembly. C5aR1 engagement by C5a stimulates LTB_4_, PGE_2_ and TXB_2_ generation, which cause endothelial dysfunctions promoting vascular leakage resulting in extensive edema. In addition, the release of LTB_4_ and KC, triggered by C5aR1 signaling, may promote the attraction and activation of neutrophils into tissue, increasing MPO levels and potentiating local immunopathology (*green lines*). The systemic C5aR1 activation, stimulated by NaV sublethal dose injection, promotes moderate increase on CCL2 and IL-6 levels, as well in the neutrophil’s percentage, which could lead envenomated animals to a several physiological disbalance (*blue lines*). By coupling human whole blood *ex vivo* (*yellow lines*) approaches and a severe experimental mouse model of envenomation, it was detected that NaV promotes a hyperacute inflammatory reaction featured by intense systemic inflammatory mediators’ storm, induced by C5a-C5aR1 axis activation. This can evolve to a plethora of pathological conditions including ALI development (*red lines*) that can progress to ARDS (*red dotted lines*) and respiratory arrest, a common death cause in humans envenomated by *N. annulifera*. In addition, these inflammatory mediators can promote organ dysfunction featuring a sepsis like syndrome, which could be a death cause of the envenomated individuals (*purple dotted lines*).

In conclusion, we have shown here, for the first time, that activation of the C5a-C5aR1 axis is the main driver of the local and systemic reactions in envenomation by *N. annulifera*, a medically important snake on Sub-Saharan Africa. **Figure 9** summarizes the main findings of the current study, in which *in vitro* and *in vivo* models targeting C5a-C5aR1 signaling demonstrated that envenomation by *N. annulifera* is a harmful hyperacute inflammatory condition that predisposes individuals to circulatory dysfunctions and ALI/ARDS development. Thus, we postulate that modulation of the C5a-C5aR1 axis could improve clinical outcomes in envenomation by *N. annulifera* as well by other venomous animals in which the C5a-C5aR1 axis is activated during physiopathogenesis.

## Data Availability Statement

The original contributions presented in the study are included in the article/**Supplementary Material**. Further inquiries can be directed to the corresponding author.

## Ethics Statement

The studies involving human participants were reviewed and approved by the Human Research Ethics Committee of the Municipal Health Secretary of São Paulo. Blood samples were obtained from healthy donors after informed consent (protocol approval n˚ 974.312 and 4.309.960). The patients/participants provided their written informed consent to participate in this study. The animal study was reviewed and approved by the Institutional Animal Care and Use Committee of the Butantan Institute (protocol approval n˚ 5323120918).

## Author Contributions

Conceived and designed the experiments: FS and DVT. Performed the experiments: FS and IV-B. Analyzed the data: FS, BC and DVT. Contributed with reagents/materials/analysis tools: DVT, BC, JL, ES, TMW. Wrote the paper: FS and DVT. All authors contributed to the article and approved the submitted version.

## Funding

This work was supported by São Paulo Research Foundation (FAPESP) funding to the Centre of Toxins, Immune Response and Cell Signalling (CeTICS) [grant 2013/07467-1]. DVT is a recipient of the CNPq Research Productivity Fellowship (grant 301358/2017-6).

## Conflict of Interest

JL is the founder of Amyndas Pharmaceuticals, which is developing complement inhibitors for therapeutic purposes. JL is inventor of patents or patent applications that describe the use of complement inhibitors for therapeutic purposes, some of which are developed by Amyndas Pharmaceuticals. JL is also the inventor of the compstatin technology licensed to Apellis Pharmaceuticals (i.e., 4(1MeW)7W/POT-4/APL-1 and PEGylated derivatives such as APL-2/pegcetacoplan).

The remaining authors declare that the research was conducted in the absence of any commercial or financial relationships that could be construed as a potential conflict of interest.

## Supplementary Material

Supplementary Figure 1
*N. annulifera* venom (NaV) induces complement activation in human whole blood. NaV (3.125 to 100 µg/mL) samples were incubated with human blood samples for 30 or 60 min at 37°C, and then anaphylatoxin generation **(A, B)** and sTCC assembly **(C)** were quantified by CBA and ELISA. Data are means ± SEM of three independent experiments. *** (different venom concentrations *vs*. saline)/### (30 *vs*. 60 minutes) p ≤ 0.001 (two-tailed two-way ANOVA, followed by Bonferroni post-test).

Supplementary Figure 2
*N. annulifera* venom (NaV) triggers lipid mediators release in human whole blood. Human blood samples were incubated with NaV (3.125 to 100 µg/mL) during 30 or 60 min at 37°C. Leukotriene B4 (LTB4) **(A)**, prostaglandin E2 (PGE2) **(B)** and thromboxane A2 (TXA2) **(C)** production were analyzed by ELISA. Data are means ± SEM of three independent experiments. *** (different venom concentrations *vs*. saline)/### (30 *vs*. 60 minutes) p ≤ 0.001 (two-tailed two-way ANOVA, followed by Bonferroni post-test).

Supplementary Figure 3
*N. annulifera* venom (NaV) upregulates chemokines generation in human whole blood. Human blood samples were exposed to NaV (3.125 to 100 µg/mL) during 30 or 60 min at 37°C. Then, CCL2, CCL5 and CXCL8 chemokines release were accessed by CBA. Data are means ± SEM of three independent experiments with different whole blood donors. *** (different venom concentrations *vs*. saline)/### (30 *vs*. 60 minutes) p ≤ 0.001 (two-tailed two-way ANOVA, followed by Bonferroni post-test).

Supplementary Figure 4 Inhibition of C5a-C5aR1 signaling worsens production of inflammatory mediators in mice injected with a lethal dose of *N. annulifera* venom (NaV). Mice (n=6/group) were pretreated with PMX205 (2 mg/kg), a C5aR1 inhibitor, or vehicle 24 and 1 hour before NaV injection. Following the inhibitor administration, severe systemic reactions were induced by the injection of NaV lethal dose, *via* intraperitoneal route. Fiver hours after envenomation, blood samples were obtained to determine systemic increase on CCL2, IL-6, IL-10, TNF-α levels by ELISA and CBA. Data are means ± SEM of six independent experiments. *** (NaV + treatments *vs*. Saline)/### (NaV + Vehicle *vs*. NaV + PMX205). p ≤ 0.001 (two-tailed t-test or two-way ANOVA, followed by Bonferroni post-test).

Supplementary Figure 5 SDS=PAGE original gels from which **Figure 2** was organized.
