# Supplementary figures and images for: C5a-C5aR1 Axis Activation Drives Envenomation Immunopathology by the Snake Naja annulifera
Source: Front Immunol. 2021 Apr 15;12:652242. doi: 10.3389/fimmu.2021.652242 (PMC8082402; doi:10.3389/fimmu.2021.652242)

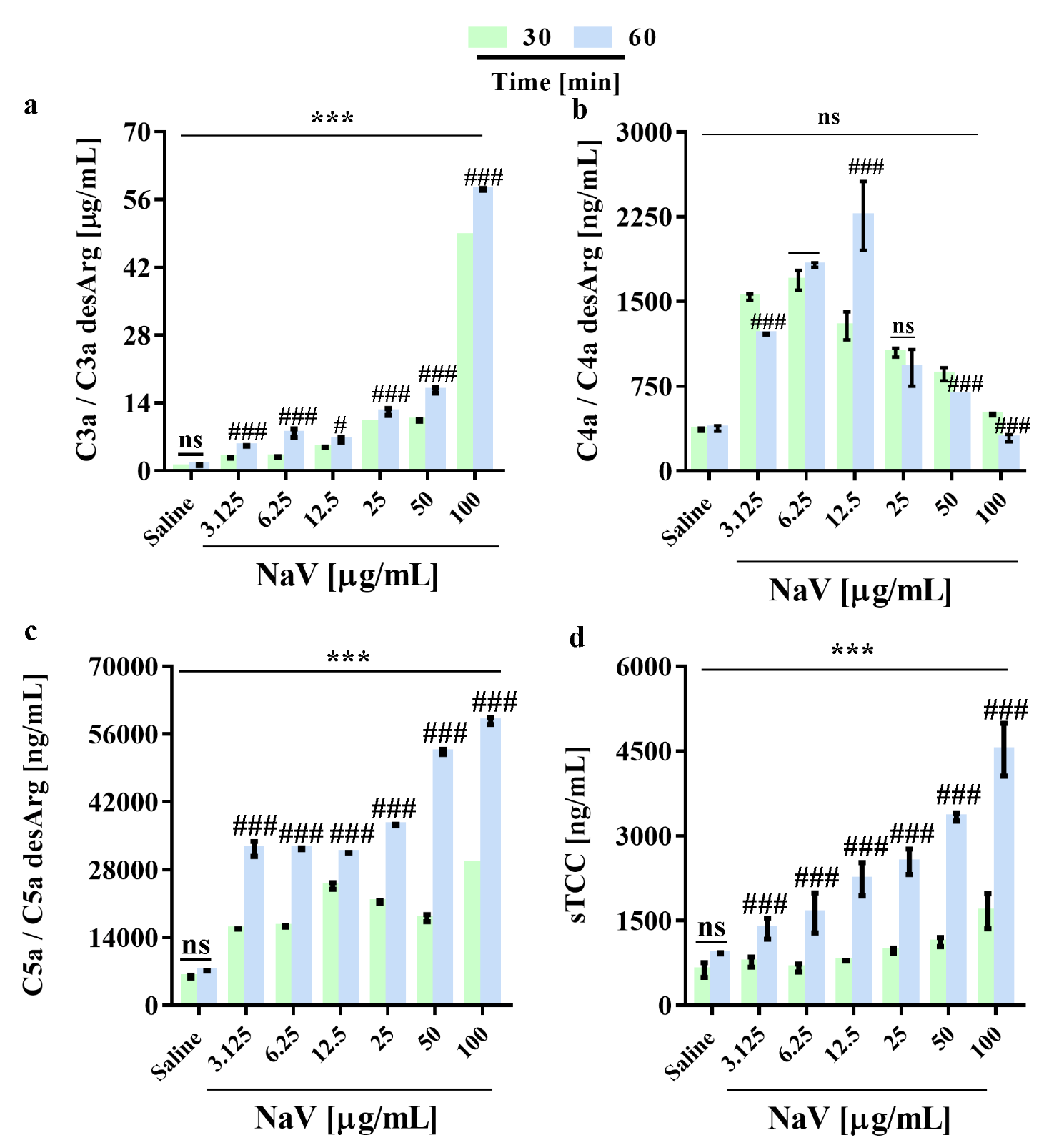

Supplement: Supplementary Figure 1 — N. annulifera venom (NaV) induces complement activation in human whole blood. NaV (3.125 to 100 µg/mL) samples were incubated with human blood samples for 30 or 60 min at 37°C, and then anaphylatoxin generation (A, B) and sTCC assembly (C) were quantified by CBA and ELISA. Data are means ± SEM of three independent experiments. *** (different venom concentrations vs. saline)/### (30 vs. 60 minutes) p ≤ 0.001 (two-tailed two-way ANOVA, followed by Bonferroni post-test). [file Image_1.tif]

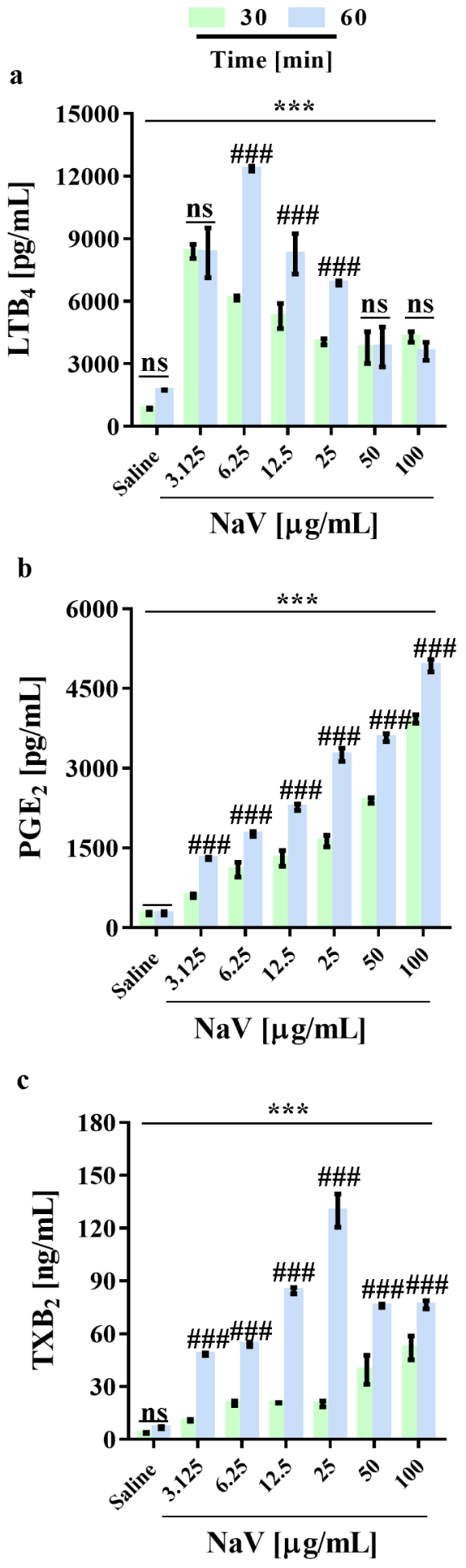

Supplement: Supplementary Figure 2 — N. annulifera venom (NaV) triggers lipid mediators release in human whole blood. Human blood samples were incubated with NaV (3.125 to 100 µg/mL) during 30 or 60 min at 37°C. Leukotriene B4 (LTB4) (A), prostaglandin E2 (PGE2) (B) and thromboxane A2 (TXA2) (C) production were analyzed by ELISA. Data are means ± SEM of three independent experiments. *** (different venom concentrations vs. saline)/### (30 vs. 60 minutes) p ≤ 0.001 (two-tailed two-way ANOVA, followed by Bonferroni post-test). [file Image_2.tif]

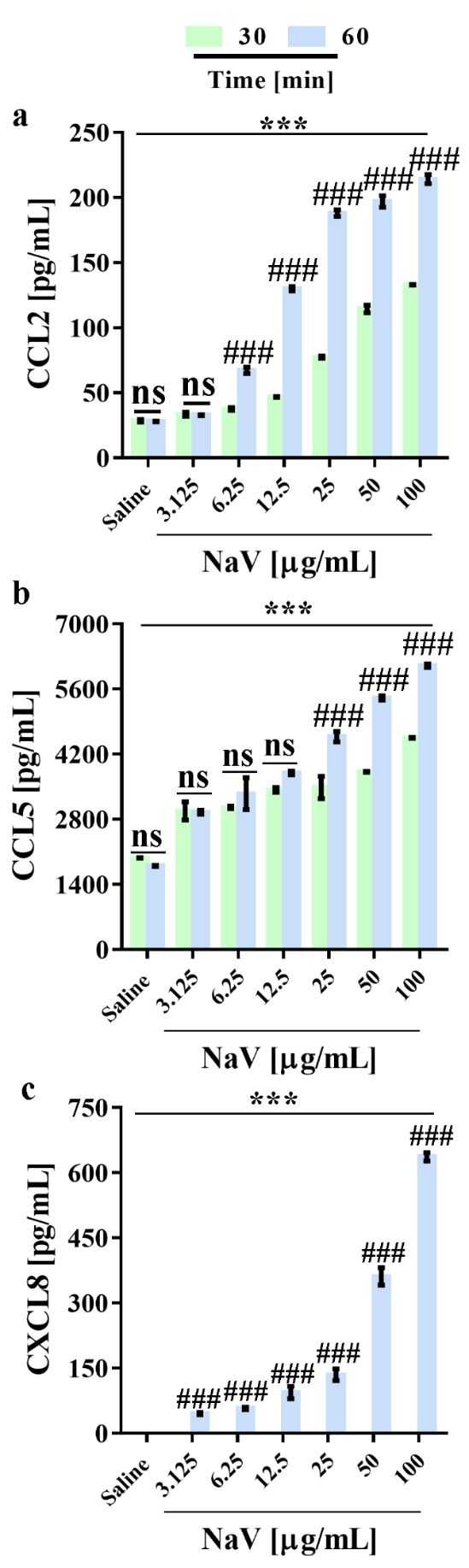

Supplement: Supplementary Figure 3 — N. annulifera venom (NaV) upregulates chemokines generation in human whole blood. Human blood samples were exposed to NaV (3.125 to 100 µg/mL) during 30 or 60 min at 37°C. Then, CCL2, CCL5 and CXCL8 chemokines release were accessed by CBA. Data are means ± SEM of three independent experiments with different whole blood donors. *** (different venom concentrations vs. saline)/### (30 vs. 60 minutes) p ≤ 0.001 (two-tailed two-way ANOVA, followed by Bonferroni post-test). [file Image_3.tif]

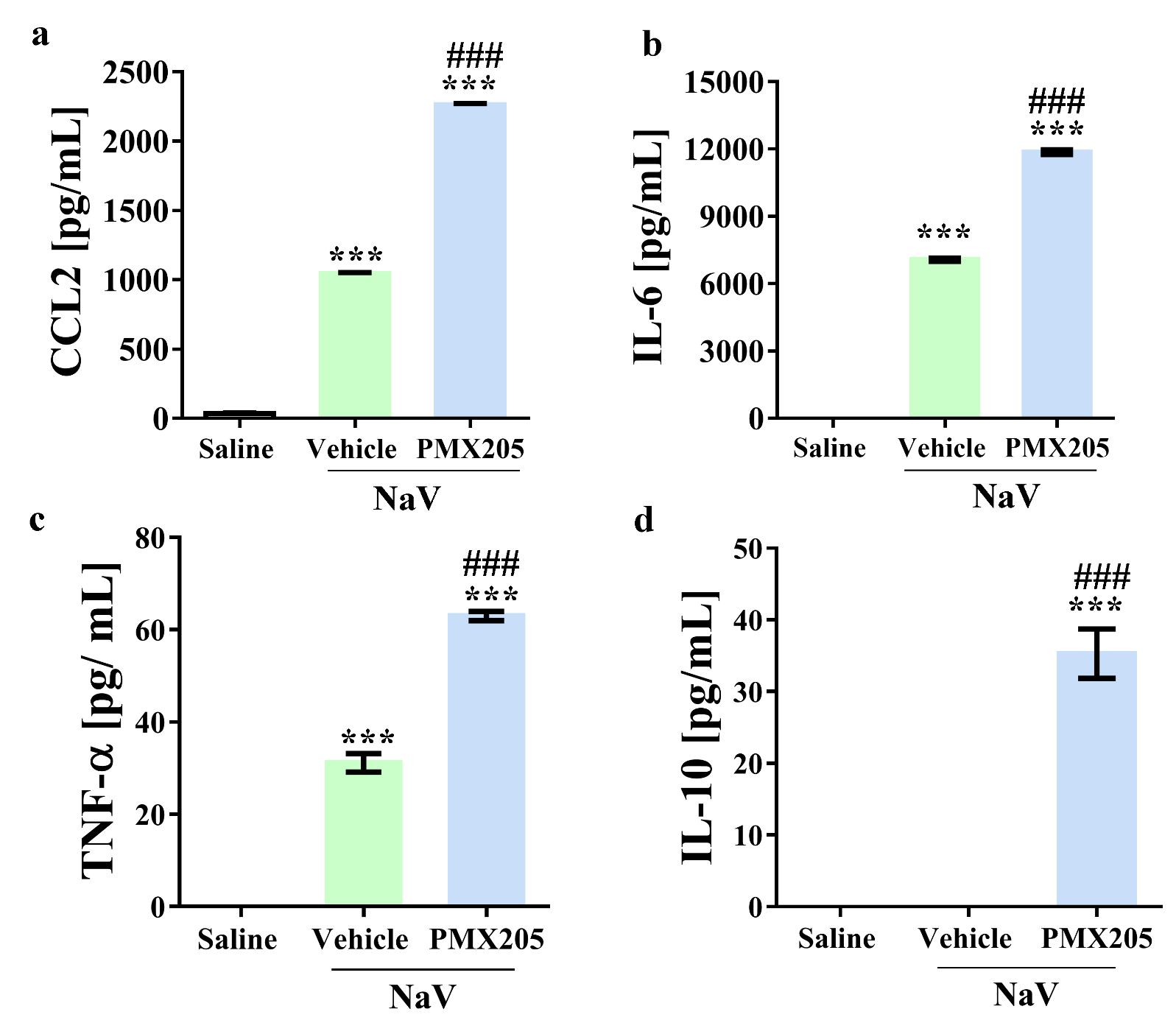

Supplement: Supplementary Figure 4 — Inhibition of C5a-C5aR1 signaling worsens production of inflammatory mediators in mice injected with a lethal dose of N. annulifera venom (NaV). Mice (n=6/group) were pretreated with PMX205 (2 mg/kg), a C5aR1 inhibitor, or vehicle 24 and 1 hour before NaV injection. Following the inhibitor administration, severe systemic reactions were induced by the injection of NaV lethal dose, via intraperitoneal route. Fiver hours after envenomation, blood samples were obtained to determine systemic increase on CCL2, IL-6, IL-10, TNF-α levels by ELISA and CBA. Data are means ± SEM of six independent experiments. *** (NaV + treatments vs. Saline)/### (NaV + Vehicle vs. NaV + PMX205). p ≤ 0.001 (two-tailed t-test or two-way ANOVA, followed by Bonferroni post-test). [file Image_4.tif]
