# Supplementary material for: C5a-C5aR1 Axis Activation Drives Envenomation Immunopathology by the Snake Naja annulifera
Source: Front Immunol. 2021 Apr 15;12:652242. doi: 10.3389/fimmu.2021.652242 (PMC8082402; doi:10.3389/fimmu.2021.652242)
Supplement: Supplementary Figure 5 — SDS=PAGE original gels from which Figure 2 was organized. [file Image_5.pdf]

C3 cleavage

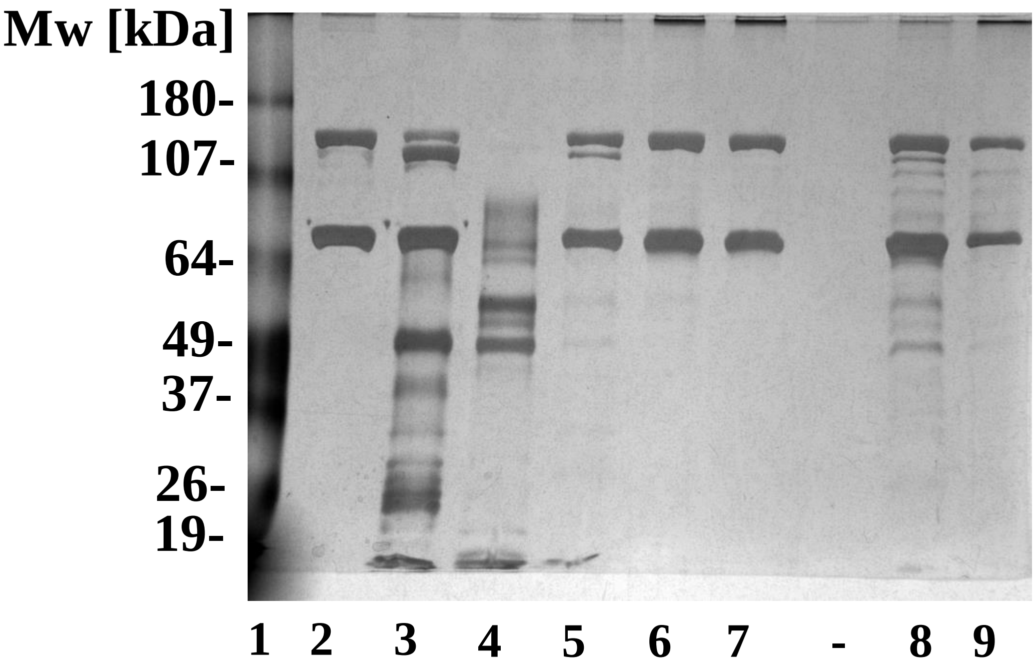

Samples
1- Molecular weight
2- C3 + vehicle (saline)
3- C3 + BjV
4- NaV
5- C3 + NaV
6- C3 + NaV + 1,10 Phe
7- C3 + 1,10 Phe
8- C3 + NaV + PMSF
9- C3 + PMSF
- Without sample

C4 cleavage

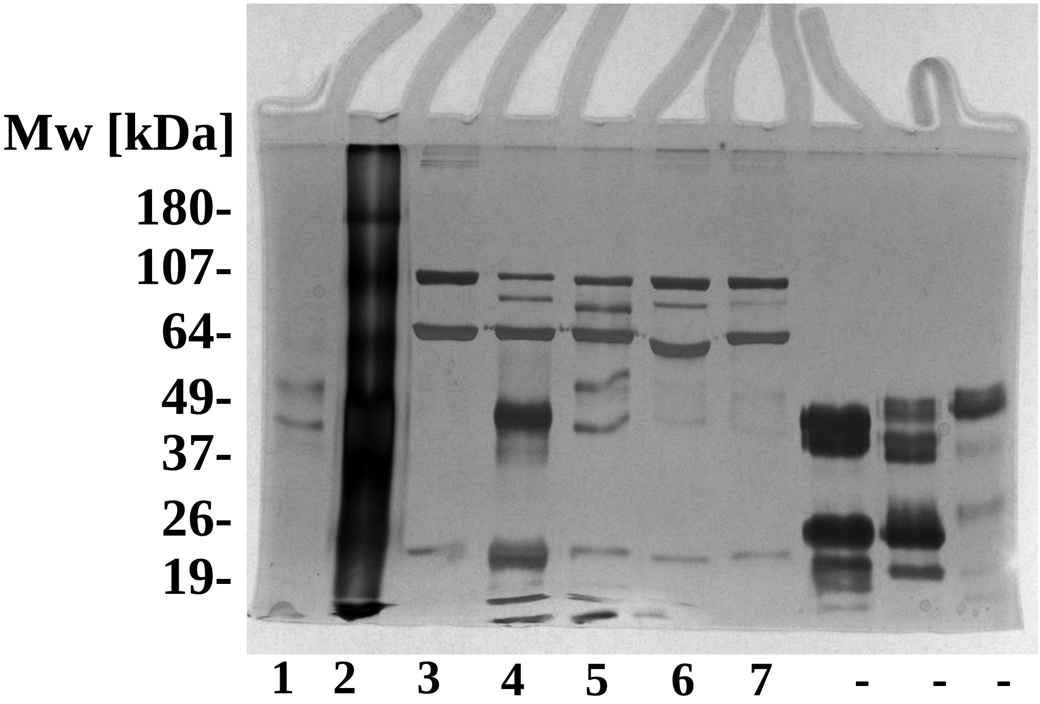

Samples

1- NaV

2- Molecular weight

3- C4 + vehicle

4- C4 + BjV

5- C4 + NaV

6- C4 + NaV + 1,10 Phe

7- C4 + NaV + PMSF

- Nonrelated samples

C5 cleavage

Mw [kDa]
180-
115-
64-
49-
37-
26-
19-

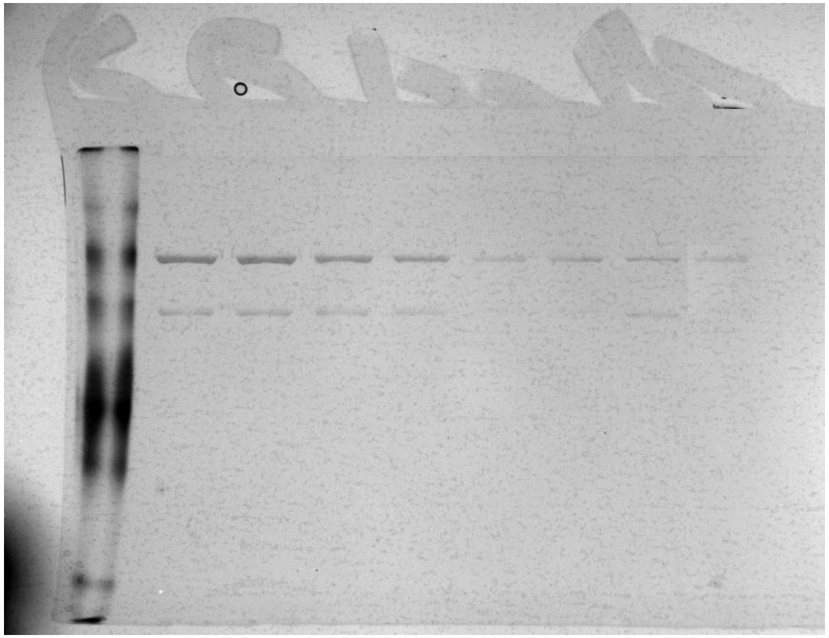

1 2 3 4 5 6 7 8 9 10

Samples

1- Molecular weight
2- C5 + vehicle (saline)
3- C5 + vehicle (ethanol)
4- C5 + NaV
5- C5 + NaV + vehicle (ethanol)
6- C5 + NaV + 1,10 Phe
7- C5 + NaV + 1,10 Phe
8- C5 + NaV + PMSF
9- C5 + NaV + PMSF
